# Supplementary material for: Simultaneous interpreting with auto-subtitling: Investigating viewer cognitive effort, stress, and comprehension
Source: PLoS One. 2025 Aug 22;20(8):e0330692. doi: 10.1371/journal.pone.0330692 (PMC12373249; doi:10.1371/journal.pone.0330692)
Supplement: S1 File — (DOCX) [file pone.0330692.s001.docx]

Questionnaire

Introduction

Dear Participant,

Please answer the questions based on your comprehension of the video presentation.

1. What is the purpose of holding this summit?

A) To promote global economic recovery

B) To emphasize national sovereignty and integrity

C) To enhance economic cooperation among countries

D) To reshape the multilateral system

2. Which principle must be ensured as a priority at this summit?

A) Promoting cultural diversity across nations

B) Emphasizing economic independence within nations

C) Respecting the purposes and principles of the Charter and international law

D) Supporting global technological innovation cooperation

3. What concept must participating nations acknowledge?

A) Economic partnerships

B) A community with a shared future

C) Cultural diversity

D) Mutual political trust

4. Why does Tunisia advocate for a future digital compact?

A) To create a safer world for information sharing

B) To improve global financial security

C) To advance the digital economy

D) To foster international collaboration in technology

5. What is the core requirement of the summit regarding the future declaration?

A) Active participation and rigorous implementation

B) Promoting regional technological collaboration

C) Strengthening international trade development

D) Facilitating resource sharing among nations

6. What is critical to achieving a transformation in the multilateral system?

A) Economic support from the international community

B) Political will and execution by nations

C) Widespread application of technological innovation

D) Enhanced communication among countries

7. Which countries are required to fulfill climate commitments?

A) Developing countries

B) High carbon-emitting countries

C) Developed countries

D) Emerging economies

8. What issues require assistance for developing countries?

A) Accelerating economic growth

B) Addressing debt challenges

C) Improving international trade capacity

D) Tackling climate-related problems

9. What commitment did parties agree upon at the previous summit?

A) Supporting global sustainable development

B) Increasing international aid efforts

C) Promoting social equity and inclusion

D) Leaving no one behind

10. What outcome is expected from this summit?

A) Launching a new phase of multilateralism rooted in shared responsibilities and global solidarity

B) Promoting fair competition and mutual respect internationally

C) Driving sustainable economic growth and development

D) Achieving equitable global resource distribution
