# Supplementary material for: Simultaneous interpreting with auto-subtitling: Investigating viewer cognitive effort, stress, and comprehension
Source: PLoS One. 2025 Aug 22;20(8):e0330692. doi: 10.1371/journal.pone.0330692 (PMC12373249; doi:10.1371/journal.pone.0330692)
Supplement: Attachment — Submitted filename: PONE-D-25-04673_reviewer.pdf [file pone.0330692.s004.pdf]

PLOS ONE

Simultaneous interpreting with auto-subtitling: investigating viewers' cognitive effort, stress, and comprehension

--Manuscript Draft--

**Manuscript Number:**	PONE-D-25-04673
**Article Type:**	Research Article
**Full Title:**	Simultaneous interpreting with auto-subtitling: investigating viewers' cognitive effort, stress, and comprehension
**Short Title:**	Comparing viewers' cognitive processing of simultaneous interpreting and auto-subtitling
**Corresponding Author:**	Andrew K.F. Cheung<br>HK PolyU: The Hong Kong Polytechnic University<br>Kowloon, HONG KONG
**Keywords:**	Auto-subtitles, Simultaneous Interpreting, Cognitive Effort, Viewing Experience, Comprehension, Electroencephalography, EEG
**Abstract:**	Simultaneous interpreting (SI) allows real-time cross-language communication without significant delays and is vital for fast-paced environments such as multilingual conferences. Automatic subtitling (auto-subtitling), facilitated by artificial intelligence (AI), is a critical mode of audiovisual translation, has been deployed by different virtual conferencing platforms to help users overcome language barriers. While both SI and auto-subtitling are essential for promoting accessibility, their impact on users' cognitive and emotional processing remains underexplored. This study evaluated the effectiveness, as well as the cognitive and emotional responses of Mandarin Chinese-speaking participants while they watched a video presentation in Arabic, a language they did not understand. Participants were divided into three groups: Group A relied on auto-subtitles in simplified Chinese characters, Group B relied on SI in Mandarin, and Group C used a combination of both methods. Analysis of electroencephalographic data and the results of comprehension tests revealed no statistically significant differences in content comprehension across the groups. However, Group A reported the poorest viewing experience, with the highest stress levels, while Group B expended the greatest cognitive effort. Group C exhibited the lowest levels of cognitive effort and stress, underscoring the advantages of dual-modality systems. These findings suggest that combining accurate auto-subtitles with professional interpreting may enhance accessibility, reduce cognitive demands, and improve the viewing experience, offering valuable insights into the integration of AI-driven technologies in SI.
**Order of Authors:**	Yanlin Li<br>Jiawen Diao<br>Andrew K.F. Cheung
**Additional Information:**	
**Question**	**Response**
**Financial Disclosure**<br><br>Enter a financial disclosure statement that describes the sources of funding for the work included in this submission. Review the submission guidelines for detailed requirements. View published research articles from *PLOS ONE* for specific examples.	Yes

Powered by Editorial Manager® and ProduXion Manager® from Aries Systems Corporation

This statement is required for submission and **will appear in the published article** if the submission is accepted. Please make sure it is accurate.<br><br>**Funded studies**<br>Enter a statement with the following details:<br>• Initials of the authors who received each award<br>• Grant numbers awarded to each author<br>• The full name of each funder<br>• URL of each funder website<br>• Did the sponsors or funders play any role in the study design, data collection and analysis, decision to publish, or preparation of the manuscript?<br><br>Did you receive funding for this work?	
Please add funding details.<br>as follow-up to "**Financial Disclosure**<br><br>Enter a financial disclosure statement that describes the sources of funding for the work included in this submission. Review the submission guidelines for detailed requirements. View published research articles from *PLOS ONE* for specific examples.<br><br>This statement is required for submission and **will appear in the published article** if the submission is accepted. Please make sure it is accurate.<br><br>**Funded studies**<br>Enter a statement with the following details:<br>• Initials of the authors who received each award<br>• Grant numbers awarded to each author<br>• The full name of each funder<br>• URL of each funder website<br>• Did the sponsors or funders play any role in the study design, data collection and analysis, decision to publish, or preparation of the manuscript?<br><br>Did you receive funding for this work?"	We would like to thank the Research and Innovation Office of the Hong Kong Polytechnic University for supporting the project (Project Code: RKQY). This manuscript was also partly supported by funding from Project number P0046386 of the Department of Chinese and Bilingual Studies of the Hong Kong Polytechnic University.

Powered by Editorial Manager® and ProduXion Manager® from Aries Systems Corporation

Please select the country of your main research funder (please select carefully as in some cases this is used in fee calculation).<br>as follow-up to "**Financial Disclosure**<br><br>Enter a financial disclosure statement that describes the sources of funding for the work included in this submission. Review the submission guidelines for detailed requirements. View published research articles from *PLOS ONE* for specific examples.<br><br>This statement is required for submission and **will appear in the published article** if the submission is accepted. Please make sure it is accurate.<br><br>**Funded studies**<br>Enter a statement with the following details:<br>• Initials of the authors who received each award<br>• Grant numbers awarded to each author<br>• The full name of each funder<br>• URL of each funder website<br>• Did the sponsors or funders play any role in the study design, data collection and analysis, decision to publish, or preparation of the manuscript?<br><br>Did you receive funding for this work?"	HONG KONG - HK
**Competing Interests**<br><br>Use the instructions below to enter a competing interest statement for this submission. On behalf of all authors, disclose any competing interests that could be perceived to bias this work—acknowledging all financial support and any other relevant financial or non-financial competing interests.<br><br>This statement is required for submission and **will appear in the published article** if the submission is accepted. Please make sure it is accurate and that any funding	The authors have declared that no competing interests exist.

sources listed in your Funding Information later in the submission form are also declared in your Financial Disclosure statement.<br><br>View published research articles from *PLOS ONE* for specific examples.<br><br>**NO authors have competing interests**<br><br>Enter: *The authors have declared that no competing interests exist.*<br><br>**Authors with competing interests**<br><br>Enter competing interest details beginning with this statement:<br><br>*I have read the journal's policy and the authors of this manuscript have the following competing interests: [insert competing interests here]*<br><br>* typeset	
**Ethics Statement**<br><br>Enter an ethics statement for this submission. This statement is required if the study involved:<br><br>• Human participants<br>• Human specimens or tissue<br>• Vertebrate animals or cephalopods<br>• Vertebrate embryos or tissues<br>• Field research<br><br>Write "N/A" if the submission does not require an ethics statement.<br><br>General guidance is provided below. Consult the submission guidelines for detailed instructions. **Make sure that all information entered here is included in the Methods section of the manuscript.**	Approved by: PolyU Institutional Review Board<br>Approval number: HSEARS20240529005<br>Oral consents were obtained.

**Format for specific study types**<br><br>**Human Subject Research (involving human participants and/or tissue)**<br>• Give the name of the institutional review board or ethics committee that approved the study<br>• Include the approval number and/or a statement indicating approval of this research<br>• Indicate the form of consent obtained (written/oral) or the reason that consent was not obtained (e.g. the data were analyzed anonymously)<br><br>**Animal Research (involving vertebrate animals, embryos or tissues)**<br>• Provide the name of the Institutional Animal Care and Use Committee (IACUC) or other relevant ethics board that reviewed the study protocol, and indicate whether they approved this research or granted a formal waiver of ethical approval<br>• Include an approval number if one was obtained<br>• If the study involved *non-human primates*, add *additional details* about animal welfare and steps taken to ameliorate suffering<br>• If anesthesia, euthanasia, or any kind of animal sacrifice is part of the study, include briefly which substances and/or methods were applied<br><br>**Field Research**<br><br>Include the following details if this study involves the collection of plant, animal, or other materials from a natural setting:<br>• Field permit number<br>• Name of the institution or relevant body that granted permission	
**Data Availability**<br><br>Authors are required to make all data underlying the findings described fully available, without restriction, and from the time of publication. PLOS allows rare exceptions to address legal and ethical concerns. See the PLOS Data Policy and FAQ for detailed information.	No - some restrictions will apply

A Data Availability Statement describing where the data can be found is required at submission. Your answers to this question constitute the Data Availability Statement and **will be published in the article**, if accepted.<br><br>**Important:** Stating 'data available on request from the author' is not sufficient. If your data are only available upon request, select 'No' for the first question and explain your exceptional situation in the text box.<br><br>Do the authors confirm that all data underlying the findings described in their manuscript are fully available without restriction?	
**Describe where the data may be found in full sentences. If you are copying our sample text, replace any instances of XXX with the appropriate details.**<br><br>• If the data are **held or will be held in a public repository**, include URLs, accession numbers or DOIs. If this information will only be available after acceptance, indicate this by ticking the box below. For example: *All XXX files are available from the XXX database (accession number(s) XXX, XXX.)*.<br>• If the data are all contained **within the manuscript and/or Supporting Information files**, enter the following: *All relevant data are within the manuscript and its Supporting Information files.*<br>• If neither of these applies but you are able to provide **details of access elsewhere**, with or without limitations, please do so. For example:<br><br>*Data cannot be shared publicly because of [XXX]. Data are available from the XXX Institutional Data Access / Ethics Committee (contact via XXX) for researchers who meet the criteria for access to confidential data.*<br><br>*The data underlying the results presented in the study are available from (include the name of the third party*	The data underlying the results presented in the study are available from the authors upon request.

and contact information or URL).<br>• This text is appropriate if the data are owned by a third party and authors do not have permission to share the data.<br><br>* typeset	
Additional data availability information:	

Manuscript

Click here to access/download;Manuscript;caption250202-PLOS-Submission.pdf

Simultaneous interpreting with auto-subtitling: investigating viewers' cognitive effort, stress, and comprehension

Yanlin Li[1¶], Jiawen Diao[2¶], Andrew K.F. Cheung[2*]

[1]Department of Industrial and Systems Engineering, Hong Kong Polytechnic University, Kowloon, Hong Kong

[2]Department of Chinese and Bilingual Studies, Hong Kong Polytechnic University, Kowloon, Hong Kong

*Andrew K.F. Cheung

E-mail: andrew.cheung@polyu.edu.hk

Abstract

Simultaneous interpreting (SI) allows real-time cross-language communication without significant delays and is vital for fast-paced environments such as multilingual conferences. Automatic subtitling (auto-subtitling), facilitated by artificial intelligence (AI), is a critical mode of audiovisual translation, has been deployed by different virtual conferencing platforms to help users overcome language barriers. While both SI and auto-subtitling are essential for promoting accessibility, their impact on users' cognitive and emotional processing remains underexplored. This study evaluated the effectiveness, as well as the cognitive and emotional responses of Mandarin Chinese-speaking participants while they watched a video presentation in Arabic, a language they did not understand. Participants were divided into three groups: Group A relied on auto-subtitles in simplified Chinese characters, Group B relied on SI in Mandarin, and Group C used a combination of both methods. Analysis of electroencephalographic data and the results of comprehension tests revealed no statistically significant differences in content comprehension across the groups. However, Group A reported the poorest viewing experience, with the highest stress levels, while Group B expended the greatest cognitive effort. Group C exhibited the lowest levels of cognitive effort and stress, underscoring the advantages of dual-modality systems. These findings suggest that combining accurate auto-subtitles with professional interpreting may enhance accessibility, reduce cognitive demands, and improve the viewing experience, offering valuable insights into the integration of AI-driven technologies in SI.

Keywords: Auto-subtitles, Simultaneous Interpreting, Cognitive Effort, Viewing Experience, Comprehension, Electroencephalography, EEG

Introduction

The purpose of this research is to experimentally test the effects of three different modes of translation—subtitles, simultaneous interpreting (SI), and a combination of both subtitles and SI—on viewer comprehension, cognitive load and viewing experience during multilingual presentations. Specifically, the study aims to assess whether same language auto-subtitles generated by artificial intelligence (AI) can facilitate content comprehension comparably to SI, to analyze the differences in cognitive demands experienced by viewers across three experimental conditions, and to identify the most effective mode to facilitate cross-linguistic communication. By systematically exploring these aspects, this research seeks to provide insights that can

enhance the understanding and engagement of audiences in multilingual settings.

Subtitles are one of the most widely adopted modes of audiovisual translation, enabling individuals to comprehend video content delivered in a foreign language. Originally developed to enhance accessibility for individuals with language barriers, subtitles have since evolved into a versatile tool with applications in education, ~~entertainment~~, and entertainment. Recent advancements in AI technologies, particularly automatic speech recognition (ASR), have revolutionized subtitling by enabling the creation of real-time automatic subtitles (auto-subtitles). Platforms such as zoom now offer auto-subtitling solutions that are fast, scalable, and cost-effective, making them accessible to global users. Despite these innovations, however, the application and effectiveness of AI-driven auto-subtitling systems remain underexplored in specialized, high-stakes settings such multilingual conferences SI is normally provided.

SI is a cornerstone of multilingual communication, facilitating communication across linguistic boundaries in international and institutional contexts. Traditionally, SI relies on highly skilled human interpreters, who deliver real-time renditions from the source language into the target language. While effective, professional SI services are resource-intensive and may not always meet the growing demand for scalable and accessible multilingual solutions. The pandemic-driven shift to online conferences has only magnified this issue [1, 2], highlighting the need for ~~innovative~~ solutions that can complement or enhance human interpreting. Recent advancements in auto-subtitling systems represent a potential solution, offering real-time textual support for audiences and addressing accessibility challenges in multilingual conferences.

Research has demonstrated the pros and cons of subtitles in different contexts. Captions, also known as same-language subtitles, and subtitles could help viewers visualize auditory content, by bridging gaps between listening and reading comprehension [3, 4]. Additionally, subtitles could improve viewers immersion and engagement by making content more accessible and predictable [5, 6]. Subtitles also help viewers maintain focus and connect with the content [7]. However, subtitles are not without limitations. Relying solely on subtitles can lead to cognitive challenges, such as increased mental effort due to the sequential processing of text and visuals [8, 9].

The dual-modality approach of providing both SI and auto-subtitles may reduce viewers' cognitive load by distributing information across multiple sensory channels. However, the dual-modality approach also requires viewers to divide their focus between reading subtitles and listening to SI. Additionally, auto-subtitles often lack the accuracy and contextual nuance of subtitles by human translators, reducing their utility in professional settings. These complexities underscore the need for empirical studies, which are currently lacking, to evaluate the interplay between multiple modalities.

This gap in the literature highlights the need for a rigorous investigation into the combined effect of auto-subtitling and SI on viewers. Such research has the potential to uncover whether this dual-modality approach enhances comprehension and accessibility or introduces additional challenges for viewers. By systematically exploring these issues, we aim to provide empirical evidence to inform both theory and practice in the fields of audiovisual translation, interpreting, and multilingual communication. Three key research questions (RQs) guide this investigation:

RQ 1: Can auto-subtitles facilitate content comprehension to the same extent as SI services?

RQ 2: Do auto-subtitles and SI impose different cognitive demands on viewers?

RQ 3: What is the most effective mode for delivering multilingual conference content to viewers?

Our study makes two significant contributions. First, it represents a pioneering effort to evaluate the impact of integrating auto-subtitling with SI on viewer comprehension, cognitive effort, and the viewing experience in conference settings. By addressing a critical gap in the literature, this research provides actionable insights for scholars and practitioners of audiovisual translation and interpreting. Second, we introduce an innovative methodology by employing electroencephalographic (EEG) techniques to objectively measure participants' cognitive load and well-being. This novel approach offers a nuanced understanding of how viewers respond to different audiovisual configurations, moving beyond self-reported data to provide empirical evidence of cognitive performance.

Literature review

Auto-subtitling and the viewing experience

Subtitles serve as textual representations of spoken information in videos, fulfilling a variety of essential functions. Initially created to provide translations by human translators for movies and television audiences facing language barriers, subtitles have evolved into indispensable tools for language learning, cognitive load management, and improving viewer comprehension [8, 10-12]. In addition, same-language subtitles serve a different purpose: they usually appear in the same language as the video's soundtrack, ensuring accessibility for Deaf and hard-of-hearing individuals.

Auto-subtitles are generated by ASR technology that enables the transcription of spoken language into text by analyzing the shape of speech waves [13]. Unlike

traditional subtitles, auto-subtitles are generated in real time, adapting to the speaker's pace and often employing innovative formats such as multi-line stacking to improve readability [14]. Auto-subtitling has gained traction for its efficiency and scalability, particularly in environments requiring immediate content delivery, such as live broadcasts, online learning platforms, and virtual meetings.

ASR has undergone significant advancements since its early stages. Davis et al. [15] pioneered early speech recognition systems capable of recognizing digits, achieving notable accuracy despite technological limitations at the time. Modern ASR systems, powered by AI and neural network architectures such as the Transformer model, have since transformed speech-to-text technology, enabling real-time transcription of large-scale audio data with increased accuracy and reliability [16]. However, challenges remain in achieving high accuracy in real-world environments, where factors such as background noise, accents, dialects, and linguistic variations complicate recognition tasks [17]. These limitations are particularly critical in high-stakes settings such as live conferences, where erroneous recognition could lead to miscommunication or misunderstandings.

Despite these challenges, auto-subtitles have become an essential tool for facilitating accessibility. They are especially valuable for ensuring accessibility for Deaf or hard of hearing individuals, as well as for native [18] and non-native speakers [19], including those in noisy or quiet environments, multitaskers, and others with specific needs or preferences. Platforms such as Zoom and MS Teams have embraced automatic subtitling as a low-cost, efficient means of providing accessibility at scale, demonstrating the versatility of ASR technology in a wide range of applications [20]. Auto-subtitling might not be as effective as having SI because it lacks the nuances of spoken language that help keep people interested and oriented [21]. Therefore, some may prefer overcoming language barriers by listening to SI because the need to expend excessive cognitive effort to follow visually a narrative may result in a negative viewing experience [22].

Research to date has primarily focused on the effect of auto-subtitles on interpreters themselves. While discussions about auto-subtilling have primarily focused on how it can assist interpreters, such as enhancing interpreters' accuracy [23], listeners could also benefit from auto-subtitling. Specifically, the auto-subtitling of an interpreter's oral rendition into written text enables listeners to visually access the speaker's message in their language. However, insufficient attention has been paid to the potential benefits of combining auto-subtitles with SI for conference attendees, rather than interpreters. This highlights the necessity of balancing visual and auditory inputs to create a holistic viewing experience. Such considerations are particularly relevant in conference settings, where attendees often include individuals with diverse

language backgrounds and cognitive abilities.

Measuring cognitive effort and viewing experience using EEG data

As auto-subtitling systems become increasingly prevalent across various contexts, understanding their impact on viewers' cognitive performance and comprehension is crucial to optimize communication and reduce language barriers [9]. The concepts of cognitive effort and stress may appear similar but are in fact distinct: cognitive effort is typically defined as the resources allocated for the processing of a task [24], while stress is a particular relationship between an individual and the environment that is appraised by the individual as taxing or exceeding their resources and endangering their well-being [25]. In the context of viewers' audiovisual content comprehension, cognitive effort refers to the mental resources required to process information while viewing auto-subtitles in the target language or simultaneously listening [9]. Meanwhile, stress may occur when the individual feels that the transmission of audiovisual information transmission exceeds their ability to cope or comfort level. This leads to an increase level of cognitive effort and stress which was used in this study as an objective measure of viewers' experience in the context of audiovisual content comprehension. Viewers' cognitive performance in audiovisual content comprehension, particularly when facilitated by auto-subtitles, represents a vital area of research that reflects the interplay between ~~technology~~ and cognitive processes.

To effectively measure cognitive effort and stress, researchers have typically employed subjective or objective assessment methods. Subjective measures, such as self-report questionnaires, allow participants to describe their perceived cognitive effort and stress levels during specific tasks [26, 27]. For instance, research participants' self-reported data in [28] indicated a reduction in cognitive load when ASR transcripts and machine-translated renditions were displayed to them. However, [29] conflict matrix model, which lacks "reliable empirical data to substantiate" [29], predicted that SI with text would have a higher cognitive load than SI without text. One of the reasons of these conflicting findings could be attributed to the limited accuracy of subjective measures.

Objective measures offer a detailed understanding of cognitive effort and stress by tracking real-time physiological responses linked to cognitive processes. These responses usually involve the central and peripheral nervous systems [30]. The most common way to accurately decode the rhythm of the nervous system is to use EEG data [31], which enables researchers to correlate specific brain responses with performance metrics. In recent years, advancements in human–computer interaction and the

increased accessibility of low-cost consumer-grade EEG devices have led to widespread recognition within research communities that objective EEG data provide a robust and reliable means of measuring participants' cognitive performance and the user experience [32-35].

While significant progress has been made in areas such as audiovisual translation and interpreting, the combined effect of auto-subtitling systems and SI on viewers remains unclear. This knowledge gap is particularly relevant given the increasing integration of advanced technologies in interpreting settings. Furthermore, traditional approaches rely heavily on subjective measures, such as user surveys and self-reported feedback; the field currently lacks robust objective techniques for assessing cognitive performance in real time. Addressing this gap is critical to advance theoretical understanding of interpreting practices and develop evidence-based methods to improve interpreting performance and user satisfaction in multilingual communication settings.

To this end, we investigated different audiovisual translation modes for the Chinese-language output of an Arabic-language conference. We evaluated the impact of these modes on viewers' content comprehension, cognitive effort, and stress to assess the feasibility of using AI-assisted auto-subtitles to enhance the delivery of conference content in the target language. To enhance the robustness of our data analysis, we incorporated EEG data as an objective measurement tool for assessing cognitive effort and stress.

Examining cognitive effort allowed us to gain valuable insights into the interplay between auditory interpretation and visual text presentation, which are essential for informing best practices in the design and implementation of auto-subtitling technology in multilingual environments. We sought to gain a comprehensive understanding of how auto-subtitling systems influence viewers' cognitive effort, comprehension and the viewing experience in the context of SI by combining neuropsychological insights with statistical techniques. Ultimately, we hope that maximizing the benefits of auto-subtitling systems while minimizing the potential cognitive strain on viewers will enhance overall comprehension and content delivery effectiveness in SI.

Method

Participants

A total of 45 participants took part in the study. All participants were native speakers of Mandarin Chinese and reported no visual impairments, hearing

impairments, or language disorders. They were recruited from various academic disciplines. Eligibility was confirmed using screening questions, including self-reported assessments of native language proficiency and visual–auditory health.

The 45 participants were randomly allocated to three distinct groups to investigate the effects of different content delivery methods on cognitive effort, viewing experience, and comprehension. Each group consisted of 15 participants, ensuring an equal distribution across the experimental conditions: Group A auto-subtitles, Group B SI, and Group C a combination of subtitles and SI. The aim of this random allocation process was to minimize bias and ensure that each group was representative of the overall participant population, allowing for a robust comparison of the effectiveness of each content delivery method.

Table 1 presents the demographic characteristics of the participants, including their age and gender, and the number of participants in each experimental group. The formal experiment commenced on November 1 and concluded on November 18, 2024. All participants wore EEG devices while watching the conference video in individual rooms without any intervention. We monitored their EEG signals from a separate, independent room. The study received ethical approval from the ethics committee of our university, with all data collected being highly protected and kept confidential.

The participants were fully informed of the study’s aims and procedures and their rights through an information sheet. Written informed consent was obtained from all of the participants, who were assured of their right to withdraw from the study at any time without consequence. They were also informed that all data would be treated with strict confidentiality.

Table 1. Demographic characteristics of participants (N = 45).

Demographic Characteristics	Total (N = 45)	Group A	Group B	Group C
**Total**	45	15	15	15
**Gender**				
Female	28	10	9	
Male	17	5	6	6
**Average Age**	23.7	23.5	23.6	23.9

EEG headset and key brain waves

The Emotiv EPOC X 14-channel wireless EEG headset was used to record brain activity, with raw EEG data accessed through the Emotiv Pro Suite installed on an HP Pavilion Plus laptop. The headset was paired with Emotiv Pro via Bluetooth, enabling effective real-time monitoring of EEG data. The Emotiv headset provides the power of five frequency bands, namely theta (4–8 Hz), alpha (8–12 Hz), low beta L (12–16 Hz), high beta H (16–25 Hz), and gamma (25–45 Hz), for each sensor, as shown in Fig 1.

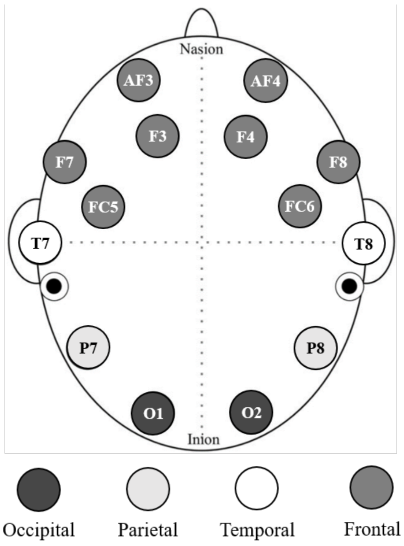

(a)

**Brain Wave**	**Frequency**	**Location**
Delta	0.5-3 Hz	Frontal region
Theta	4-7 Hz	Parietal and temporal regions
Alpha	8-13 Hz	Occipital region
Beta	14-30 Hz	Parietal and frontal regions

(b)

Fig 1. Electrode placement of the Emotiv Epoc X EEG headset. AF: anterior–frontal, F: frontal, FC: fronto-central, T: temporal, P: parietal, O: occipital. The odd numbers denote the left hemisphere and even numbers represent the right hemisphere. Black dots indicate locations of reference electrodes.

Analyzing brain wave patterns is essential for understanding cognitive effort and stress levels. While the relationship between alpha wave fluctuations and cognitive workload remains somewhat unclear [36], neural oscillations associated with theta and beta waves are recognized as reliable markers of cognitive workload [37]. According to previous studies [30, 38], theta waves (4–8 Hz) are associated with memory processing and cognitive load; increased theta activity may indicate significant cognitive effort and greater strain on working memory [38] and increased mental fatigue [39-41]. In such a setting, theta power can indicate mental resource allocation during multimodal (audiovisual) content processing. Higher theta activity in the frontal region corresponds to executive functions, including decision-making, working memory, and semantic processing, while occipital region activity reflects visual processing and reading comprehension [42]. Regarding beta waves, Lazarus and Folkman [43] identified elevated beta wave power as an indicator of increased stress levels, as beta waves are associated with heightened cognitive activity and emotional strain during task performance. In particular, high beta wave activity often reflects mental states characterized by tension, anxiety, or demands associated with sustaining attention under pressure. These findings are further supported by research in interpreting and language translation contexts, which has used beta wave activity to measure cognitive and emotional strain [39, 40].

Materials

A speech in Arabic from a United Nations (UN) conference was selected as the experimental material due to UN's practice of providing SI services into six official languages, aligning with our focus on evaluating comprehension of SI in professional settings. For UN open meetings that provide SI services, video recordings as well as audio recordings in both source language target languages could be downloaded from its website. Chinese was chosen as the target language, namely the language into which the speech was interpreted and subtitled. Three versions of the same speech were prepared for the experiment:

Version A: Chinese auto-subtitling generated by an ASR system based on the Chinese SI.

Version B: Chinese SI.

Version C: A combination of Chinese auto-subtitling and Chinese SI.

The speech content, pacing, and audiovisual quality were identical across the three versions, with each video lasting for 3 minutes and 40 seconds. This controlled experimental design ensured that the only independent variable was the mode of conference content delivery (auto-subtitling, SI, or a combination of both), with Chinese as the target language in all conditions.

To assess the participants' comprehension of the speech in the target language, a multiple-choice test was developed in simplified Chinese characters. The test consisted of 10 questions focused on factual recall, such as the speech's main topics, numerical data, and named entities, to evaluate the participants' immediate understanding of the ~~interpreted~~ content. An example of a question translated in English is as follows:

"What is the purpose of holding this summit?

A) To promote global economic recovery

B) To emphasize national sovereignty and integrity

C) To enhance economic cooperation among countries

D) To reshape the multilateral system"

Each correct answer was awarded 10 point, with a total possible score of 100 points. To ensure the test's validity and reliability, all questions and answer options were reviewed by a linguistics expert who specialized in translation and interpreting. This review ensured that the questions aligned with the speech content and that the wording of the answer options was clear, precise, and appropriate for assessing comprehension of the target language.

Experimental procedure

A pilot test was conducted prior to the main study to ensure the feasibility of the experimental design. This included evaluating the functionality of video playback, the clarity of the instructions, and the timing of the comprehension test. Feedback from the pilot phase informed minor adjustments, such as rewording comprehension questions to improve clarity and ensuring the smooth operation of all technical components. The pilot confirmed that the materials and procedures were ready for the main experiment.

The experiment was conducted in a controlled computer lab environment to minimize external distractions and ensure consistent testing conditions. The participants were randomly assigned to one of the three conditions using a computer-generated randomization sequence, ensuring unbiased allocation.

The experimental procedure is depicted in Fig 2. Upon arrival, the participants were provided with a consent form to read and sign, ensuring ethical compliance with the study protocol. Following this, participants were briefed on the experimental procedure and introduced to the wireless EEG device, which was used to monitor the participants' cognitive activity. A research assistant then helped the participants to put on the wireless EEG headset. The research team ensured that the EEG device had achieved 100% contact quality before proceeding. Once the EEG setup was complete, the participants were seated at individual stations to view their assigned video and complete the comprehension test. During the video viewing, we monitored the participants from a separate room to ensure that the EEG data were collected without interruptions or technical issues. The environment was controlled to eliminate distractions, such as noise or external interference, and thereby maintain consistent conditions across the participants.

Each participant individually viewed their assigned video, which lasted for 3 minutes and 40 seconds. To maintain consistency, the video could not be paused or replayed during the experiment. Immediately after viewing the video, the participants completed an online comprehension test on a computer. The comprehension questions were only accessible after the video had ended, to ensure that the participants relied solely on the presented material.

Throughout the experiment, we monitored the EEG signals in real time to track the participants' cognitive activity. The EEG system provided wireless, non-invasive monitoring, ensuring the participants' comfort and freedom of movement during the video viewing and test phases. After completing the experiment, each participant received a HK$50 gift card as compensation for their time and participation. Fig 2 illustrates the complete experimental procedure. Fig 3 shows photographs of two participants wearing EEG devices while watching the video material, demonstrating the

experimental setup in action.

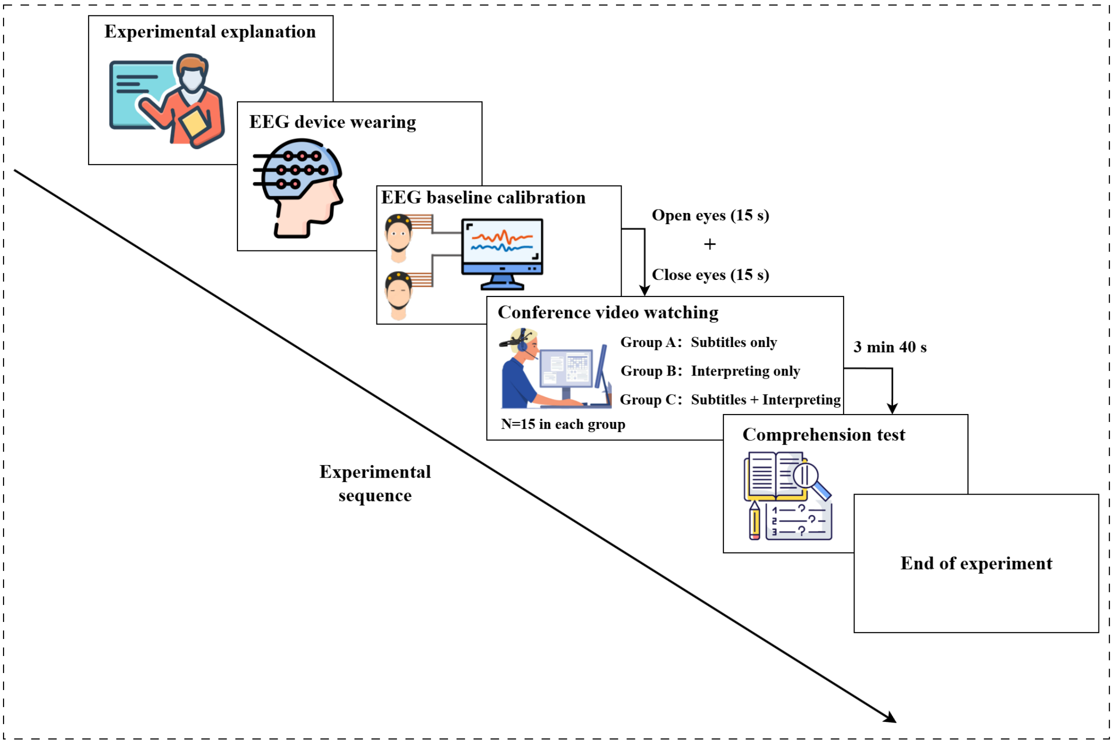

Fig 2. Experimental procedure.

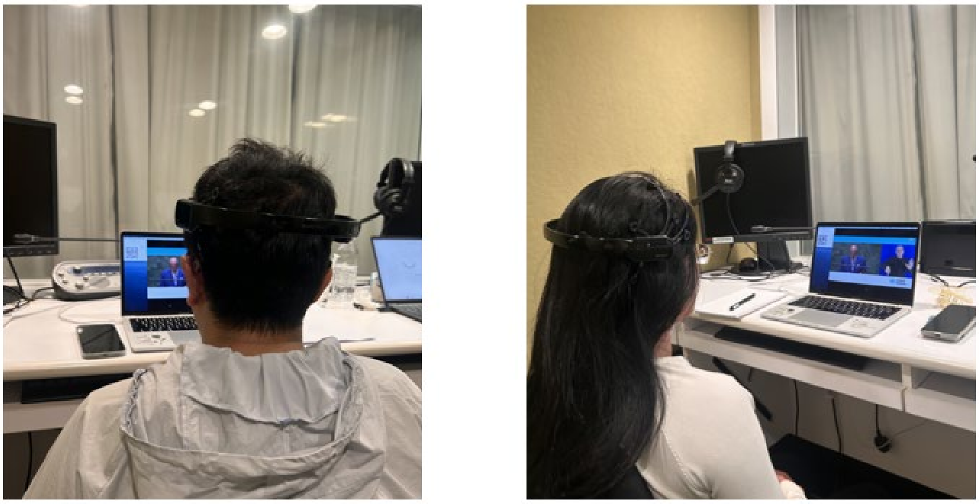

Fig 3. Participants wearing EEG devices watching the video.

Results

Content comprehension

We designed a questionnaire to test the participants' comprehension, aiming at information retrieval and free recall from the video. The participants were required to

watch the assigned video once only, without pausing or rewinding. After viewing, the participants were asked to casually comment on how they felt while watching the video. The three groups' comprehension scores are shown in Table 2.

Table 2. Comprehension scores of the three groups.

Group	Max	Min	Median	Mode	Mean	SD
Group A	100	20	80	90	70.00	23.30
Group B	100	40	85	80	74.29	15.55
Group C	100	30	85	90	75.63	18.25

Both the mean and median scores across the groups were relatively high, suggesting similar levels of comprehension across conditions. The Kruskal–Wallis test was chosen for its robustness to small samples and its ability to handle non-normally distributed data. The analysis yielded an H statistic of 0.44 ($df = 2, p = 0.803$). As $p > 0.05$, the null hypothesis was not rejected, indicating that there were no statistically significant differences in score distributions between the three conditions.

Cognitive effort and viewing experience

Participants' neurological responses to three distinct content delivery modes were investigated through analysis of brain activity patterns across participant groups. The topographical distribution of the raw EEG data was visualized using the EEGLab toolbox [44] in Matlab R2022a, as illustrated in Figs 4 and 5. The analysis revealed distinct patterns of neural activity across the three groups, with particular emphasis on power bands, which are strongly associated with human cognitive performance.

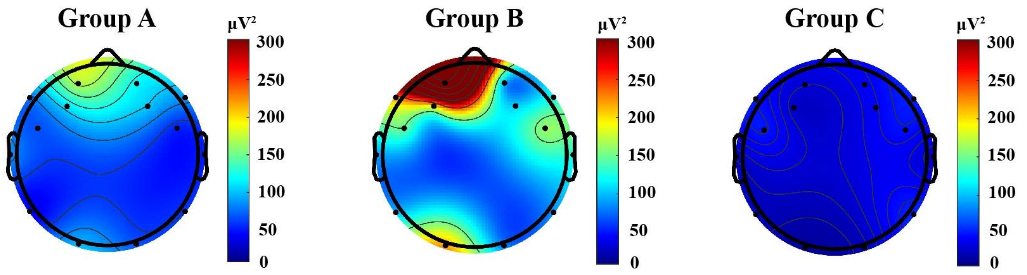

Fig 4. Topographic brain activity heatmap of EEG data in the theta band (4–8 Hz) for the three groups.

Fig 4 presents the neural oscillations associated with theta waves, which have been recognized as reliable markers of cognitive effort [37], for the three groups. Typically, an increase in theta wave power is associated with tasks involving greater cognitive effort, strain on working memory [38], and mental fatigue [41]. These findings have been supported by research in interpreting contexts [39, 40], which has suggested that theta waves indicate mental resource allocation during multimodal (audio-visual) content processing.

As shown in Fig 4, Group B demonstrated the highest theta band activation in both

the frontal region (AF3) and the occipital region (O7), followed by Group A, with lower theta band activation in the frontal region (AF3), and then Group C, with limited theta band activation.

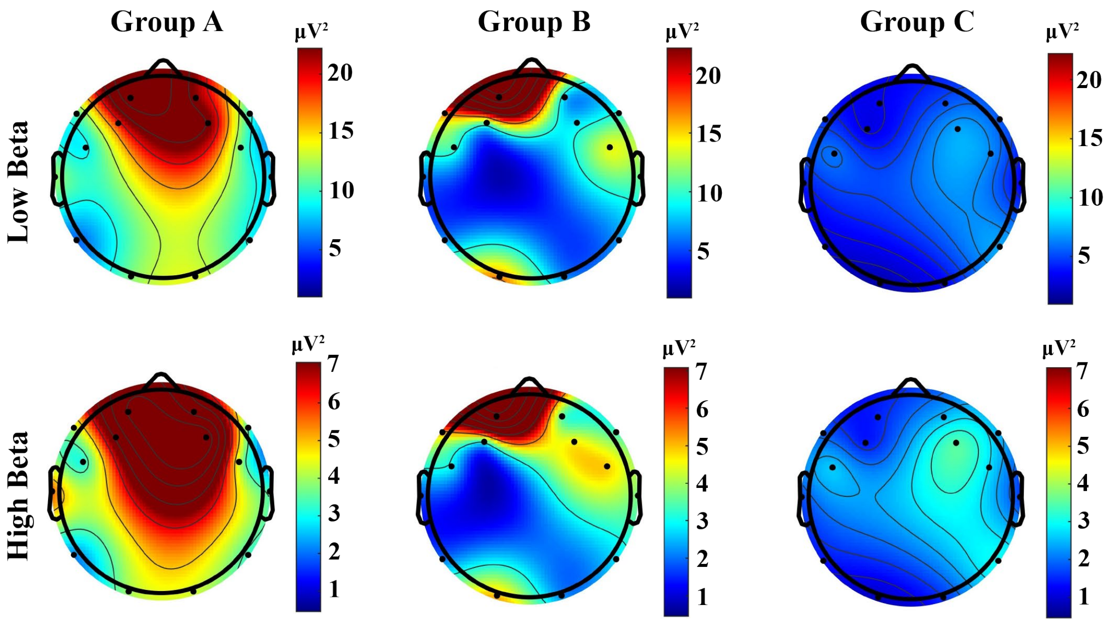

Fig 5. Topographic brain activity heatmap of EEG data in the beta bands for the three groups.

Regarding the viewing experience, Fig 5 illustrates the hierarchical pattern of beta waves associated with active cognitive processing, problem-solving, and alertness, providing insights into the participants' engagement levels and potential stress responses. Elevated beta waves activity is generally correlated with excessive levels may indicate anxiety or stress [43, 45]. Results show that Group A exhibited the highest beta band activation, indicating the highest stress level, followed by Group B, with Group C showing the lowest level of stress.

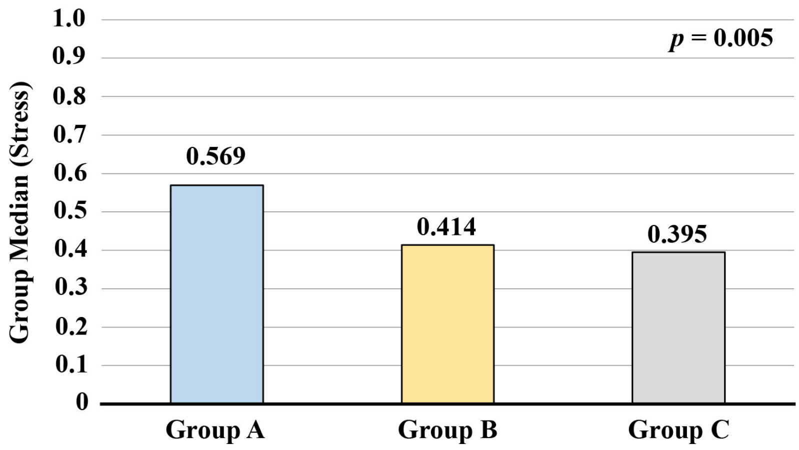

Fig 6. Non-parametric Kruskal–Wallis *H* test results (stress) for the three groups.

In addition to the raw EEG data, which were subsequently drawn as a topographic brain activity heatmap, the Emotiv Pro Suite software package was used to obtain stress values for the participants from the EEG devices while watching the 220-second

Arabic-language video. We performed the non-parametric Kruskal–Wallis H test for the three groups, and the results are shown in Fig 6. There were significant differences in the pressure values of the three groups $p < 0.01$, demonstrating the accuracy and rationality of the discrepancies in the beta band.

Discussion

Can auto-subtitles facilitate content comprehension to the same extent as SI services?

The experimental results indicated that the mode of delivery of translated information across the three groups did not significantly influence viewers' comprehension scores. This aligns with the finding of previous studies that comprehension is not markedly affected by the unavailability of audio input when subtitles are present. This suggests that subtitles alone can provide sufficient information for understanding, although potentially at the cost of a more demanding cognitive process. Other studies have further supported this observation, finding that even the non-syntactic segmentation of subtitles (i.e., splitting sentences at unnatural points) does not negatively impact viewers' comprehension [46, 47].

While our statistical analysis revealed only minor differences in comprehension scores across experimental conditions, the group exposed to a combination of auto-subtitles and SI achieved the highest average comprehension scores. This finding underscores the potential of multimodal translation delivery to enhance the viewing experience. The dual-modality approach that distributes information across auditory and visual channels reduces cognitive load and comprehension.

Do auto-subtitles and SI impose different cognitive demands on viewers?

The experimental findings yielded important insights into cognitive effort and stress patterns during conference content processing across the three experimental groups. Analysis of EEG topographic brain activity indicated significant variations in cognitive effort and stress levels between the SI (listening) and auto-subtitles (reading) conditions, offering valuable theoretical and practical implications.

The theta waves measurements demonstrated a hierarchical pattern of cognitive demand: Group B (SI only) exhibited the highest theta power, followed by Group A (auto-subtitles only), while Group C (combined SI and auto-subtitles) showed the lowest level. This finding is particularly relevant to conference interpreting, as conference attendees relying on translation services experience varying levels of cognitive load depending on the modality through which they receive translated information. The heightened cognitive effort in the SI-only condition suggests additional mental resources were needed for participants to comprehend by processing only auditory information while those listened to SI likely due to the mental effort

required to process and comprehend auditory input in real time. In contrast, attendees using auto-subtitles only showed lower cognitive demand, as reading visual text may involve a less resource-intensive processing pathway. Most notably, attendees who had access to both SI and auto-subtitles, exhibited the lowest theta power, indicating reduced cognitive strain when provided with multimodal inputs. The minimal cognitive effort observed for Group C finds robust theoretical support from the bilingual dual-coding theory [48] that suggests memory performance improves when information is processed through multiple channels, in our case the visual and audio channels. These findings extend beyond simple cognitive load measurement, suggesting that the traditional reliance on audio-only SI may not fully optimize audience comprehension and engagement.

The comparison of Groups A and B reveals an interesting deviation from conventional assumptions that watching subtitle videos without corresponding audio would increase cognitive load as viewers use metacognitive strategies to adjust their visual processing to maintain comprehension [9, 49]. Audio input is transient and sequential, while visual input is often static and simultaneous, allowing viewers to process information at their own pace and revisit it as needed. Our finding may reflect the experimental setting, which simulated a conference setting where attendees typically listen to speakers for various purposes. In this context, participants were motivated to understand the experimental material, whether through auto-subtitles, SI, or a combination of both, potentially requiring additional cognitive resources for comprehension and retention. Our findings suggest that the professional context significantly may influence how different modalities affect cognitive processing.

Beta wave analysis revealed distinct stress patterns across the three groups in relation to the viewing experience. Elevated beta waves are closely associated with stress responses, making them reliable indicators of mental workload and emotional strain [43]. Previous studies have employed beta wave analysis to evaluate user experiences in various experimental contexts, such as game design and operational platform usability [34, 35].

In our study, Group C reported the best viewing experience, characterized by the lowest beta levels, followed by Group B. Group A showed the highest beta levels and thus the poorest viewing experience suggesting the additional cognitive demands of processing complex professional content through the visual modality. Szarkowska et al. [9] noted that in the absence of sound in the first language (L1), participants often utilize metacognitive strategies, such as reading subtitles more thoroughly, to maintain comprehension.

Conversely, the lower beta wave power observed in Group B suggests a state of relative relaxation, probably due to the fluidity of the auditory input, which did not

require finding visual cues to compensate for understanding. Similarly, the lowest beta wave activity in Group C indicated that the participants experienced minimal stress.

These findings suggest that multimodal presentations not only enhance cognitive processing but also contribute to a less stressful viewing experience. The availability of both auditory and visual appears to provide the least stressful experience in Group C by presenting translated information in both modalities. The integration of SI and auto-subtitles not only enhances the accessibility of complex content but could potentially reduce stress level, enabling attendees to maintain focus and engagement throughout the event. The superior performance of the combined modality condition (Group C) indicates that conference organizers should consider providing both SI and real-time subtitles whenever feasible. Furthermore, the increased cognitive effort required for audio-only SI implies that traditional interpreting services could be enhanced by complementary visual aids, particularly at technical or otherwise specialist conferences, where terminology presents additional challenges.

What is the most effective mode for delivering multilingual conference content to viewers?

Based on our data, greater cognitive efforts for comprehension were needed for SI than for auto-subtitles or for a combination of auto-subtitling and SI. This increased cognitive burden may arise from the fleeting nature of verbal language where participants could neither control the speed of delivery nor revisit the renditions. Such limitations highlight the potential benefits of auto-subtitles, particularly for individuals who struggle with word decoding and processing speed. Moreover, it is essential to acknowledge the constraints of working memory and attention in the context of information processing [50, 51]. Viewers often face challenges in retaining and synthesizing information when it is presented rapidly. Therefore, providing a dual modality that combines SI with auto-subtitles may enhance understanding and retention.

Additionally, captions provide access to audio content accessibility to the Deaf or and hard of hearing individuals. This inclusivity is crucial in fostering equitable access to information, particularly in educational and professional settings. The integration of visual and auditory elements via this dual-modality approach not only provides less stressful experience but also promotes engagement among diverse viewers.

Therefore, the combination of auto-subtitles and SI could be the most effective strategy for communication in multilingual conferences. This dual approach alleviates cognitive effort and stress by leveraging the complementary strengths of both modalities: auto-subtitles provide a visual aid for decoding, while SI offers auditory context and tonal nuances. This redundancy in information delivery may reduce the strain on working memory, leading to a less stressful experience for attendees with varying levels of proficiency in the source language.

Practical implications

The findings of this study highlight several practical implications for reducing cognitive load, and enhancing the usability of audiovisual content, particularly in the context of SI.

Our findings indicate that participants experience reduced stress level when both real-time auto-subtitles and SI are available. This dual-modality approach supports individuals who struggle with word decoding, have limited working memory, or face challenges in processing spoken information due to fast speech rates or complex terminology. Auto-subtitles provide participants with visual support to follow along in real time. This is particularly valuable for individuals with attention limitations, as it provides additional input to supplement the way they process information. By reducing cognitive load and stress, this approach improves the overall viewing experience and ensures that content is accessible to a wider range of users.

Moreover, our experiment of the dual-modality approach offers valuable insights for the field of education, particularly in online and remote learning environments. Students in multilingual classrooms or those learning in non-native languages can benefit greatly from having both visual (L1 subtitles) and auditory (L1 interpreting) aids. This approach facilitates deeper comprehension by enabling students to follow along at their own pace, revisit subtitles, and benefit from auditory context and intonation. Multimodal delivery is particularly useful for subjects with complex or technical content. Furthermore, captions are essential for students with auditory impairments, ensuring equitable access to online courses and digital learning platforms.

Conclusions

We investigated the combined effects of auto-subtitling and SI on viewers' comprehension, cognitive effort, and overall viewing experience in a conference interpreting setting. Using a controlled experimental design, we examined three distinct modes of delivering conference content in the target language (Mandarin Chinese): auto-subtitling, SI, and a combination of the two. The findings contribute to the expanding field of audiovisual translation and interpreting studies by offering novel insights into the potential benefits and challenges of dual-modality approaches in enhancing comprehension and accessibility. The results indicated that the integration of auto-subtitles and interpreting, compared with either modality alone, reduced viewers' cognitive effort and improved their overall viewing experience, although comprehension differences between the groups were not statistically significant.

Additionally, our analysis of EEG data suggested that participants in the SI-only condition (Group B) invested the greatest cognitive effort, whereas the subtitling-only condition (Group A) imposed a lower cognitive load. Interestingly, despite expending more cognitive effort, the participants in Group B reported a better viewing experience, appearing more relaxed than the participants in Group A.

Despite these promising results, our study has limitations, including a relatively small and homogeneous participant group; reliance on ASR-generated subtitles, which may not fully capture the nuances of SI renditions; a short speech duration; and a focus on a single language pair (Arabic to Chinese). These factors may limit the generalizability of the findings. Future research should address these issues by including larger and more diverse samples, testing longer and more complex materials, and exploring the application of dual-modality systems across different language pairs and cultural contexts. Additionally, further attention could be paid to mitigating split-attention effects, such as optimizing subtitle placement or pacing, to further enhance the user experience. Finally, applying this experimental framework in real-world conference settings could offer a more comprehensive understanding of the practicality, scalability, and effectiveness of combining auto-subtitling and SI to address the growing demand for inclusive and accessible multilingual communication solutions.

References

1. Cheung AKF. Listeners' perception of the quality of simultaneous interpreting and perceived dependence on simultaneous interpreting. Interpreting. 2022;24(1):38-58. https://doi.org/10.1075/intp.00070.che.
2. Cheung AKF. Cognitive load in remote simultaneous interpreting: Place name translation in two mandarin variants. Humanities and Social Sciences Communications. 2024;11(1):1238. https://doi.org/10.1057/s41599-024-03767-y.
3. Garza TJ. Evaluating the use of captioned video materials in advanced foreign language learning. Foreign Language Annals. 1991;24(3):239-58. https://doi.org/10.1111/j.1944-9720.1991.tb00469.x.
4. Danan M. Captioning and subtitling: Undervalued language learning strategies. Meta: Journal des Traducteurs/Translators' Journal. 2004;49(1):67-77. https://doi.org/10.7202/009021ar.
5. Guillory HG. The effects of keyword captions to authentic french video on learner comprehension. CALICO Journal. 1998;15(1-3):89. PMID: 750440780.
6. van der Zee T, Admiraal W, Paas F, Saab N, Giesbers B. Effects of subtitles, complexity, and language proficiency on learning from online education videos. Journal of Media Psychology: Theories, Methods, and Applications. 2017;29(1):18-30.

https://doi.org/10.1027/1864-1105/a000208 PMID: 1883359401; 2017-13880-003.
7. Kruger J-L, Doherty S, Soto-Sanfiel M-T. Subtítulos en lengua original: Sus efectos en el espectador nativo y extranjero/ original language subtitles: Their effects on the native and foreign viewer. Comunicar. 2017;25(50):23-32. Epub Spanish ed. https://doi.org/10.3916/C50-2017-02 PMID: 1880253417.
8. Liao SX, Kruger JL, Doherty S. The impact of monolingual and bilingual subtitles on visual attention, cognitive load, and comprehension. Journal of Specialised Translation. 2020;(33):70-98. PMID: WOS:000532624400004.
9. Szarkowska A, Ragni V, Szkriba S, Black S, Orrego-Carmona D, Kruger J-L. Watching subtitled videos with the sound off affects viewers' comprehension, cognitive load, immersion, enjoyment, and gaze patterns: A mixed-methods eye-tracking study. PLoS One. 2024;19(10):e0306251. https://doi.org/10.1371/journal.pone.0306251.
10. Baranowska K. Learning most with least effort: Subtitles and cognitive load. ELT Journal. 2020;74(2):105-15. https://doi.org/10.1093/elt/ccz060.
11. Díaz-Cintas J. The name and nature of subtitling. In: Bogucki Ł, Deckert M, editors. The palgrave handbook of audiovisual translation and media accessibility. Cham: Springer International Publishing; 2020. p. 149-71.
12. O'Sullivan C, Cornu JF. *History of audiovisual translation*. The routledge handbook of audiovisual translation. 2019, New York: Routledge.
13. Alharbi S, Alrazgan M, Alrashed A, Alnomasi T, Almojel R, Alharbi R, et al. Automatic speech recognition: Systematic literature review. IEEE Access. 2021;9:131858-76. https://doi.org/10.1109/ACCESS.2021.3112535.
14. Cintas JD, Remael A. *Audiovisual translation: Subtitling*. 2007, London: Routledge.
15. Davis KH, Biddulph R, Balashek S. Automatic recognition of spoken digits. The Journal of the Acoustical Society of America. 1952;24(6):637-42. https://doi.org/10.1121/1.1906946.
16. Zhang Q, Lu H, Sak H, Tripathi A, McDermott E, Koo S, et al., *Transformer transducer: A streamable speech recognition model with transformer encoders and rnn-t loss*. in *2020 IEEE International Conference on Acoustics, Speech and Signal Processing (ICASSP)*. 2020. p. 4-8.
17. Fujimoto M, Kawai H, *One-pass single-channel noisy speech recognition using a combination of noisy and enhanced features*. in *Interspeech Conference*, Int Speech Commun A, editors. 2019, Graz, Austria.
18. Negi S, Mitra R. Native language subtitling of educational videos: A multimodal analysis with eye tracking, eeg and self-reports. British Journal of Educational Technology. 2022;53(6):1793-816. https://doi.org/10.1111/bjet.13214.
19. d'Ydewalle G, Praet C, Verfaillie K, Rensbergen JV. Watching subtitled television:

Automatic reading behavior. Communication Research. 1991;18(5):650-66. https://doi.org/10.1177/009365091018005005.

20. Malakul S, Park I. The effects of using an auto-subtitle system in educational videos to facilitate learning for secondary school students: Learning comprehension, cognitive load, and satisfaction. Smart Learning Environments. 2023;10(1):4. https://doi.org/10.1186/s40561-023-00224-2.

21. Gorman BM, Crabb M, Armstrong M. *Adaptive subtitles: Preferences and trade-offs in real-time media adaption*. in *Proceedings of the 2021 CHI Conference on Human Factors in Computing Systems*, 2021, Yokohama, Japan: Association for Computing Machinery. p. 733.

22. Becerra H, Ragano A, Debnath D, Ullah A, Lucas CR, Walsh M, et al. Dialogue understandability: Why are we streaming movies with subtitles? PMID: INSPEC:25017235.

23. Böser U, Deane-Cox S. *Translation, interpreting and technological change : Innovations in research, practice and training*. 2024, London: Bloomsbury Publishing.

24. Paas FGWC. Training strategies for attaining transfer of problem-solving skill in statistics: A cognitive-load approach. Journal of Educational Psychology. 1992;84(4):429-34. https://doi.org/10.1037/0022-0663.84.4.429.

25. Folkman S. Stress: *Appraisal and coping*. Encyclopedia of behavioral medicine. 2020, Cham: Springer International Publishing.

26. Thoen A, Steyaert J, Alaerts K, Evers K, Van Damme T. A systematic review of self-reported stress questionnaires in people on the autism spectrum. Review Journal of Autism and Developmental Disorders. 2023;10(2):295-318. https://doi.org/10.1007/s40489-021-00293-4.

27. Westbrook A, Braver TS. Cognitive effort: A neuroeconomic approach. Cognitive, Affective, & Behavioral Neuroscience. 2015;15(2):395-415. https://doi.org/10.3758/s13415-015-0334-y.

28. Chen S, Kruger J-L. The effectiveness of computer-assisted interpreting. Translation and Interpreting Studies. 2023;18(3):399-420. https://doi.org/10.1075/tis.21036.che.

29. Seeber KG. *Multimodal processing in simultaneous interpreting*. The handbook of translation and cognition. 2017.

30. Das Chakladar D, Roy PP. Cognitive workload estimation using physiological measures: A review. Cognitive Neurodynamics. 2024;18(4):1445-65. https://doi.org/10.1007/s11571-023-10051-3.

31. Aghajani H, Garbey M, Omurtag A. Measuring mental workload with eeg+fnirs. Frontiers in Human Neuroscience. 2017. https://doi.org/10.3389/fnhum.2017.00359 PMID: 2289583692.

32. Cano S, Araujo N, Guzman C, Rusu C, Albiol-Pérez S. Low-cost assessment of user experience through eeg signals. IEEE Access. 2020;8:158475-87. https://doi.org/10.1109/ACCESS.2020.3017685.

33. Li T, Chmiel A. Automatic subtitles increase accuracy and decrease cognitive load in simultaneous interpreting. Interpreting. 2024;26(2):253-81. https://doi.org/10.1075/intp.00111.li.

34. Li Y, Po Tsang Y, Lee CKM, Han S. Integrating neurophysiological sensing and group-based multi-criteria decision-making for fourth-party logistics platform selection. Advanced Engineering Informatics. 2025;64:102968. https://doi.org/10.1016/j.aei.2024.102968.

35. Paranthaman PK, Bajaj N, Solovey N, Jennings D, *Comparative evaluation of the eeg performance metrics and player ratings on the virtual reality games*. in *2021 IEEE Conference on Games (CoG)*, 2021, p. 17-20.

36. Castro-Meneses LJ, Kruger JL, Doherty S. Validating theta power as an objective measure of cognitive load in educational video. Educational Technology Research and Development. 2020;68(1):181-202. https://doi.org/10.1007/s11423-019-09681-4 PMID: WOS:000513352400009.

37. Puma S, Matton N, Paubel PV, Raufaste É, El-Yagoubi R. Using theta and alpha band power to assess cognitive workload in multitasking environments. International Journal of Psychophysiology. 2018;123:111-20. https://doi.org/10.1016/j.ijpsycho.2017.10.004 PMID: WOS:000424175300014.

38. Klimesch W, Schack B, Sauseng P. The functional significance of theta and upper alpha oscillations. Experimental Psychology. 2005;52(2):99-108. https://doi.org/10.1027/1618-3169.52.2.99 PMID: WOS:000228121400002.

39. Boos M, Kobi M, Elmer S, Jäncke L. The influence of experience on cognitive load during simultaneous interpretation. Brain and Language. 2022;234. https://doi.org/10.1016/j.bl.2022.105185 PMID: WOS:000859473800001.

40. Grabner RH, Brunner C, Leeb R, Neuper C, Pfurtscheller G. Event-related eeg theta and alpha band oscillatory responses during language translation. Brain Research Bulletin. 2007;72(1):57-65. https://doi.org/10.1016/j.brainresbull.2007.01.001 PMID: WOS:000244820100008.

41. Tran Y, Craig A, Craig R, Chai R, Nguyen H. The influence of mental fatigue on brain activity: Evidence from a systematic review with meta-analyses. Psychophysiology. 2020;57(5):e13554. https://doi.org/10.1111/psyp.13554.

42. Isabella SL, Cheyne JA, Cheyne D. Inhibitory control in the absence of awareness: Interactions between frontal and motor cortex oscillations mediate implicitly learned responses. Frontiers in Human Neuroscience. 2021;15. https://doi.org/10.3389/fnhum.2021.786035.

43. Lazarus RS, Folkman S. *Stress, appraisal, and coping*. 1984, Springer Publishing Company.
44. Delorme A, Makeig S. Eeglab: An open source toolbox for analysis of single-trial eeg dynamics including independent component analysis. Journal of Neuroscience Methods. 2004;134(1):9-21. https://doi.org/10.1016/j.jneumeth.2003.10.009.
45. Palva S, Palva JM. New vistas for α-frequency band oscillations. Trends in Neurosciences. 2007;30(4):150-8. https://doi.org/10.1016/j.tins.2007.02.001.
46. Gerber-Morón O, Szarkowska A, Woll B. The impact of text segmentation on subtitle reading. Journal of Eye Movement Research. 2018;11(4). https://doi.org/10.16910/jemr.11.4.2 PMID: 33828704; PubMed Central PMCID: PMCPMC7901653.
47. Perego E, Del Missier F, Porta M, Mosconi M. The cognitive effectiveness of subtitle processing. Media Psychology. 2010;13(3):243-72. https://doi.org/10.1080/15213269.2010.502873 PMID: WOS:000281850100003.
48. Paivio A, Desrochers A. A dual-coding approach to bilingual memory. Canadian Journal of Psychology/Revue canadienne de psychologie. 1980;34(4):388-99. https://doi.org/10.1037/h0081101 PMID: 614291410; 1982-02758-001.
49. Schnotz W. *An integrated model of text and picture comprehension*. The cambridge handbook of multimedia learning. 2005, West Nyack: Cambridge University Press.
50. Justice LM, Lomax R, O'Connell A, Pentimonti J, Petrill SA, Piasta SB, et al. Are working memory and behavioral attention equally important for both reading and listening comprehension? A developmental comparison. Reading and Writing. 2018;31(7):1449-77. https://doi.org/10.1007/s11145-018-9840-y.
51. Mayer, R.E., *The Cambridge Handbook of Multimedia Learning*. 2nd edition ed. Cambridge Handbooks in Psychology. 2014, West Nyack: Cambridge University Press.
